# Work Loss and Income Before and After Diagnosis of Addison's Disease: A Population-Based Longitudinal Study

**DOI:** 10.1210/clinem/dgaf696

**Published:** 2025-12-30

**Authors:** Stavros Stergianos, Åsa H Everhov, Jonas Söderling, Jakob Skov, Sophie Bensing

**Affiliations:** Department of Medicine, Solna, Division of Experimental Endocrinology, Karolinska Institutet, Stockholm SE-171 76, Sweden; Department of Endocrinology, Karolinska University Hospital, Stockholm SE-171 76, Sweden; Department of Medicine, Solna, Division of Clinical Epidemiology, Karolinska Institutet, Stockholm SE-171 76, Sweden; Department of Clinical Science and Education, Södersjukhuset, Karolinska Institutet, Stockholm SE-118 83, Sweden; Department of Medicine, Solna, Division of Clinical Epidemiology, Karolinska Institutet, Stockholm SE-171 76, Sweden; Department of Molecular Medicine and Surgery, Karolinska Institutet, Stockholm SE-171 76, Sweden; Center for Clinical Research, Region Värmland, Karlstad SE-651 85, Sweden; Department of Medicine, Solna, Division of Experimental Endocrinology, Karolinska Institutet, Stockholm SE-171 76, Sweden; Department of Endocrinology, Karolinska University Hospital, Stockholm SE-171 76, Sweden

**Keywords:** Addison's disease, work loss, sick leave, disability pension, income, socioeconomics

## Abstract

**Context:**

The effect of autoimmune Addison's disease (AAD) on work ability and income over time remains unclear.

**Objective:**

This work aimed to evaluate differences and temporal trends in income and lost workdays among patients with AAD before and after diagnosis, vs matched general population comparators. This nationwide, register-based cohort study was conducted in Sweden. We linked the Swedish Addison Register with national health registers to identify 716 working-age individuals (aged 20-62 years) with incident AAD 2003 to 2019 and 3271 general-population comparators matched by sex, age, county, calendar year, and education level. Annual work loss, taxable earnings, and disposable income were examined over a 10-year period around diagnosis using linear (work loss) and quantile (earnings, income) regression.

**Results:**

Patients with AAD had significantly more work loss than comparators from 1 year before to 3 years after diagnosis, with the largest difference the year after diagnosis (mean difference: 30.5 days; 95% CI, 22.9-38.0 days). Median taxable earnings were lower in patients with AAD 1 year before (median difference: $−1107; 95% CI, $−2042 to $−173; *P* = .02) and 1 year after diagnosis ($−1105; 95% CI, $−2166 to $−43; *P* = .04). Disposable income was also reduced 1 year after diagnosis (median difference: $−1084; 95% CI, $−1813 to $−354; *P* = .004).

**Conclusion:**

In AAD, increased work loss and reduced earnings and income were concentrated around diagnosis, peaking in the year after. Reassuringly, both absence from work and income normalized within 4 years following diagnosis.

Chronic health conditions can substantially impair patients' ability to engage in and sustain employment at their full capacity. Work ability is integral to financial status and influences social, emotional, and functional aspects of life (1). Disability not only affects the individual's well-being and quality of life but also has broader socioeconomic implications, highlighting the need for targeted interventions to support affected individuals, and for welfare systems that can help mitigate these effects (2).

Autoimmune Addison's disease (AAD), the major cause of primary adrenal insufficiency in high-income countries, arises from autoimmune destruction of the adrenal cortex, impairing cortisol, aldosterone, and androgen production (3, 4). The prevalence ranges from 5 to 221 cases per million (5), and the annual incidence is approximately 4 to 6 cases per million individuals (5). AAD often coexists with other autoimmune diseases (6) like autoimmune thyroid disease, type 1 diabetes (7), gonadal failure in women, and autoimmune gastritis (5). The initial diagnosis of AAD is frequently delayed, as early symptoms are nonspecific and can mimic more common conditions (8).

Despite glucocorticoid replacement therapy, patients with AAD experience increased fatigue (9) and sleep disturbances (9, 10).Younger females may face challenges in executive functioning during everyday activities (11). Patients with AAD are at increased risk of cardiovascular diseases and infection-related hospitalizations (12-17), with gastroenteritis and other infections commonly precipitating Addisonian crises (3, 18). Comorbid conditions, particularly type 1 diabetes, further increase vulnerability (19).

Work-related difficulties are common among patients with AAD. A recent nationwide Swedish study reported a 23% work loss in 2019 (20), consistent with earlier smaller studies showing disability rates of 14% to 26% in individuals with adrenal insufficiency (20-25). However, previous studies were exclusively cross-sectional, often limited by small cohorts, mixed patient populations, or absence of national data. The added risk of diagnostic delay and uncertainty about recovery of work ability highlight the need for a longitudinal study around the time of diagnosis.

The aim of this study was to investigate differences and fluctuations in lost workdays, taxable earnings, and disposable income among working-age patients with AAD before and after diagnosis, compared to randomly sampled, matched individuals from the Swedish population.

## Materials and Methods

### Study Design

This was a Swedish, nationwide, register-based cohort study of patients with AAD and comparators from the country's general population (matched by sex, age, county of residence, calendar year, and level of education).

### Setting

Sweden has a comprehensive and inclusive social security system that offers support to its citizens (26). Despite widening income gaps (27), inequalities are still slightly below the European average (28), and low from a global perspective (29).

Employment protection is strictly regulated (30), and the country has a universal public health-care system primarily funded through taxation. During the last years of the study period, patients in Sweden paid for prescription medications up to a high-cost threshold of 2200 SEK (~$230 USD) within a 12-month period, after which they were exempt from further charges (31).

For sick leave episodes lasting up to 14 days, employers cover 80% of the employee's salary (32). For sick leave longer than 14 days, the Swedish Social Insurance Agency provides compensation through sickness benefits (“sjukpenning”), which also cover unemployed individuals and those on parental leave (33).

Individuals with a permanent work capacity reduction of at least 25% following more than 12 months of sick leave are eligible for age-based compensation. Those aged 30 to 64 years receive sickness compensation (“sjukersättning”), while those aged 19 to 29 years receive activity compensation (“aktivitetsersättning”). Both forms of compensation are typically lower than sickness benefits. In this study, we use the term “*disability pension*” to refer both to sickness and activity compensation. The standard retirement age during the study period was 65 years, though retirement could occur between 61 and 67 years under specific conditions.

### Data Sources

In Sweden, all residents are assigned a unique personal identity number (34) at birth or on immigration. This number facilitates the linkage of various national registers. For this study, we linked data from: the Swedish Addison Register (SAR), the Swedish National Patient Register (NPR), the Swedish Prescribed Drug Register (PDR), the Swedish Longitudinal Integrated Database for Health Insurance and Labor Market Studies (LISA), the Micro Data for Analysis of the Social Insurance Database (MIDAS), and the Total Population Register (TPR).

The SAR (35), a research register founded in 2008, includes clinical information and blood samples from more than half of all Swedish patients diagnosed with AAD. It is regarded as the world's largest register of its kind.

The NPR (36), established in 1964 and achieving nationwide coverage by 1987, contains data on hospital admissions, discharges, and diagnosis codes according to the International Statistical Classification of Diseases and Related Health Problems (ICD). Since 2001, the register has also included information on hospital-based outpatient care. The register's validity is considered high (36).

The PDR (37), launched in July 2005, provides comprehensive nationwide data on all dispensed prescription medications. This includes details on drug type according to the Anatomical Therapeutic Chemical (ATC) classification system, as well as information on dispensed quantity, dosage, and associated costs.

The LISA database (38), established in 1990, encompasses individuals aged 16 years or older residing in Sweden. It compiles information on sick leave, disability pensions, civil status, migration, taxable and disposable income, unemployment benefits, and various social welfare payments. Reporting to this database is mandatory.

MIDAS, a nationwide database, contains detailed records on the type and dates of social insurance benefits, including both sick leave and disability pension. All registered compensation is directly associated with work absence. Parental leave is recorded separately and excluded from the sick leave data.

The TPR (39) is a national registry that documents life events such as births, deaths, and migration. Reporting rates are high, with death data recorded within 30 days in nearly 100% of cases and migration data reported within 30 days for approximately 91% of cases.

### Patient Identification and Selection of General Population Comparators

The study sample was drawn from a data linkage consisting of a matched cohort of patients with AAD and general population comparators (matched 1:10 by age, sex, and county of residence), specifically assembled to investigate multiple aspects of AAD, as described in previous publications (20, 40).

The dataset specific to this study was constructed by identifying all individuals in the Swedish population aged 20 to 62 years at the time of a first registered diagnosis of primary adrenal insufficiency or Addisonian crisis in the NPR between January 1, 2003, and December 31, 2019. Diagnoses were identified using ICD codes version 10 (E27.1, E27.2). Previous versions 7 to 9, ICD-7 (274.4), ICD-8 (255.1), and ICD-9 (255E), were also used to exclude prevalent AAD. The date of diagnosis was defined as the date of the first recorded AAD-related diagnostic code (index date). To examine outcomes around the time of AAD diagnosis, we restricted the age range to 20 to 62 years to ensure that all individuals were adults of working age and, importantly, able to contribute at least 2 years of follow-up both before and after diagnosis while remaining within the working-age window.

Individuals in the SAR (with detectable 21-hydroxylase antibodies) who met the inclusion criteria were directly included in the study. For individuals identified through the NPR but not included in the SAR, the diagnosis of AAD was validated using the PDR, in accordance with previously established criteria (20, 40). Validation required documentation of at least 2 dispensed prescriptions for hydrocortisone (ATC: H02AB09) or cortisone acetate (ATC: H02AB10), in combination with fludrocortisone (ATC: H02AA02).

**Figure 1.**
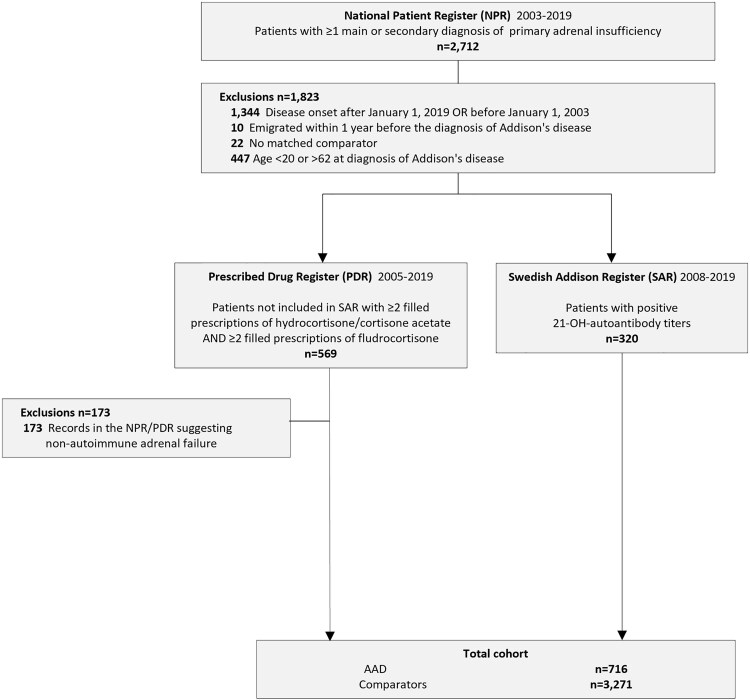
Flowchart of inclusion of study participants with incident Addison's disease (2003-2019). Abbreviations: AAD, Autoimmune Addison's disease.

Beyond the initial matching by age, sex, and county of residence, each patient with AAD was further matched to up to 5 comparators based on calendar year and educational attainment. Individuals who migrated to or from Sweden within 1 year prior to the index date were not eligible for inclusion. Exclusion criteria were applied at start of follow-up to patients identified via the NPR (but not SAR) and all the comparators to eliminate cases of adrenal insufficiency due to secondary or nonautoimmune causes (Supplementary Table S1 (41)). The same exclusion criteria were applied to comparators, and only those left after exclusions were eligible for matching. Details of the inclusion and exclusion process for the included patients with AAD are presented in Fig. 1. The final matching ratio of comparators per AAD case is detailed in Supplementary Table S2 (41). The start of follow-up was defined as the date of the first recorded AAD diagnosis (index date), which was applied consistently both to patients with AAD and their matched comparators.

### Outcomes

The primary outcomes were mean annual work loss days (measured as days of sick leave or disability pension), taxable earnings, and disposable household income. All outcomes were assessed over a 10-year period, spanning 5 years before and after the index date. Work loss was calculated relative to the date of first diagnosis of AAD, while taxable earnings and disposable income were evaluated per calendar year. In this study, the calendar year of the first registered diagnosis of AAD corresponds to year 0 to 1.

Work loss was assessed using compensation data for sick leave and disability pension. For each study participant, exact compensation dates were retrieved from MIDAS, and the annual net number of work loss days in relation to index date was calculated by weighting the compensated days according to the proportion of benefit received (25%-100%). Importantly, short-term sick leave compensated by employers (ie, sick pay) is not captured and was therefore not included in this study. Consequently, participants with zero recorded work loss days may still have experienced short-term work loss episodes (≤14 days) that were not captured in the data.

Information on taxable earnings, disposable income, educational attainment, marital status, unemployment, and retirement was obtained from the LISA database by calendar year. Taxable earnings encompass gross income from wages, including sickness benefits paid by the employer during the initial 14 days of work absence. Annual individualized household income serves as an indicator of standard of living. It was calculated by multiplying the family's disposable income by the individual's consumption weight and dividing by the family's consumption weight. Disposable income includes gross earnings including broader sources of income minus final taxes and deductions (Supplementary Table S3 (41)).

### Covariates

Data on educational attainment, marital status, retirement, and unemployment compensation were extracted from the LISA database. Education was categorized into 3 levels: primary and lower secondary education (≤9 years), upper secondary education (10-12 years), and postsecondary education (≥13 years). Information on comorbidities (Table 1) was obtained from the NPR. Type 1 diabetes was identified by at least one recorded diagnosis of ICD-10 code E10 (including all subcodes).

**Table 1.** Characteristics of incident patients (2003-2019) and matched comparators in Sweden at first registered diagnosis of autoimmune Addison's disease

Characteristics	Overall	
	AAD	Comparators
N	716	3271
**Sex, n (%)**		
Male	298 (41.6%)	1358 (41.5%)
Female	418 (58.4%)	1913 (58.5%)
**Age at diagnosis/index date, y**		
Mean (SD)	39.4 (11.7)	39.4 (11.7)
Median (25th-75th)	39 (30-49)	38 (30-48)
Range, min-max	20-62	20-62
Age groups, n (%), y		
20-39	379 (52.9%)	1735 (53.0%)
40-62	337 (47.1%)	1536 (47.0%)
**Year of diagnosis/index date**		
2003-2008	259 (36.2%)	1188 (36.3%)
2009-2014	242 (33.8%)	1100 (33.6%)
2015-2019	215 (30.0%)	983 (30.1%)
**Level of education, n (%), y**		
≤ 9	71 (9.9%)	248 (7.6%)
10-12	351 (49.0%)	1673 (51.1%)
≥ 13	292 (40.8%)	1347 (41.2%)
Missing	2 (0.3%)	3 (0.1%)
**Marital status**		
Married/registered partner	281 (39.2%)	1234 (37.7%)
**Comorbidity, n (%)**		
Ischemic heart disease	5 (0.7%)	17 (0.5%)
Cerebrovascular disease	3 (0.4%)	10 (0.3%)
Type 1 diabetes (≥1 E10 diagnosis)	87 (12.2%)	24 (0.7%)
Hypertension	48 (6.7%)	184 (5.6%)
Depression and anxiety	102 (14.2%)	435 (13.3%)
Autoimmune thyroid disease	77 (10.8%)	103 (3.1%)
Malignant neoplasms	16 (2.2%)	53 (1.6%)
**Employment status at y of diagnosis/index date, n (%)**		
Unemployment	59 (8.2%)	317 (9.7%)
Retirement	22 (3.1%)	166 (5.1%)
**Follow-up from date of diagnosis/index date, y**		
Mean (SD)	8.2 (4.9)	8.4 (4.9)
Median (25th-75th)	8.0 (4.0-12.2)	8.3 (4.2-12.4)
Range, min-max	0.0-17.0	0.0-17.0
Categories, n (%)		
<5 y	229 (32.0%)	1006 (30.8%)
≥ 5 y	487 (68.0%)	2265 (69.2%)

### Subgroup Analyses

Subgroup analyses of the outcomes were performed by sex, age group (aged 20-39 and 40-62 years), educational level (≤9, 10-12, and ≥13 years), and presence of comorbid type 1 diabetes.

### Additional Analyses

We performed an additional analysis assessing the prevalence of autoimmune thyroid disease (ICD-10: E035, E039, E050, E055, E063, E065) 5 years after the index date.

An exploratory analysis was conducted to compare time to disability pension between patients with AAD and their matched comparators, both within the 5-year study period and over an extended 15-year follow-up.

### Statistics

Work loss outcomes were reported as the mean net number of lost workdays per individual per year in relation to index date. Mean differences between patients with AAD and matched general-population comparators were estimated using linear regression. Ninety-five percent CIs were derived using nonparametric bootstrapping. In addition to mean values, the proportion of individuals with any work loss and the relative risk of experiencing work loss were also reported.

Taxable earnings and disposable income by calendar year were calculated in Swedish kronor (SEK), adjusted for inflation to the year 2024, and converted to United States dollars (USD or $) using the average annual exchange rate (1 USD = 10.5614 SEK). As the data were not normally distributed, results are presented as median values with 95% CIs. Differences in taxable earnings and disposable income between patients with AAD and their matched comparators were estimated using quantile regression, implemented via the QUANTREG procedure in SAS. 95% CIs were obtained through resampling. Time to disability pension was evaluated using Cox proportional hazards regression.

All statistical tests were 2-sided, with a statistical significance threshold set at *P* less than .05. Analyses were performed using SAS software, version 9.4 (SAS Institute Inc).

### Ethics

The study was conducted in alignment with the principles of the Declaration of Helsinki and approved by the Regional Ethics Review Board in Stockholm, Sweden (DNR 2006/026/3 and DNR 2020-04374 for linkage of SAR data). Informed consent was not required for this register-based study.

## Results

We identified 716 patients with incident AAD (58% women) and 3271 matched comparators from the general population of Sweden. The median (interquartile range) age at diagnosis (also index date) was 39 years (range, 30-49 years). The median (interquartile range) follow-up time was 8.0 years (range, 4.0-12.2 years). Sixty-eight percent of patients with AAD had at least 5 years of follow-up after diagnosis. Baseline characteristics are outlined in Table 1.

### Work Loss

**Figure 2.**
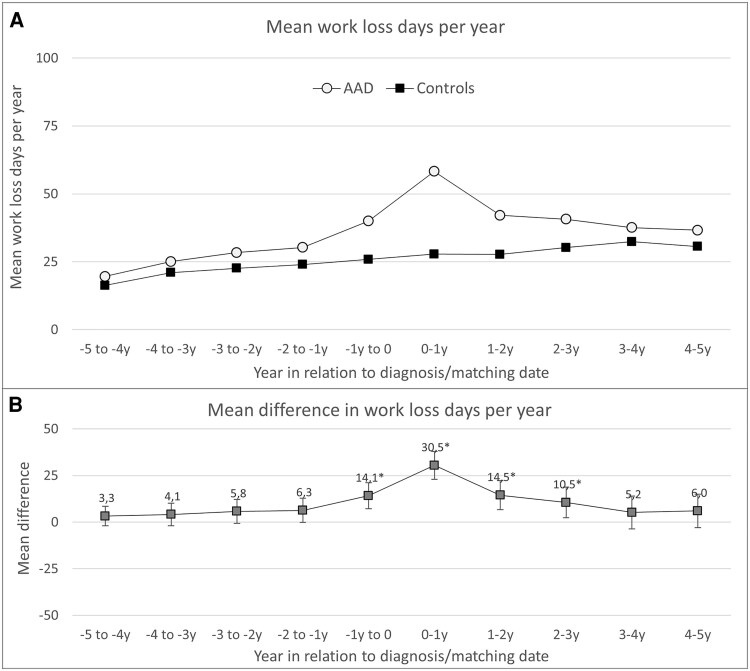
A, Mean work loss days and B, mean difference in work loss days per year, among incident patients with autoimmune Addison's disease (AAD) in Sweden between 2003 and 2019 and matched comparators, from 5 years before to 5 years after diagnosis. *P less than .05.

Patients with AAD exhibited significantly increased work loss from 1 year before to 3 years after diagnosis compared to their matched comparators. The highest observed difference occurred in the year post diagnosis, with patients experiencing an average of 30.5 additional lost workdays (95% CI, 22.9-38.0). Temporal trends in work loss among study participants are illustrated in Fig. 2.

**Figure 3.**
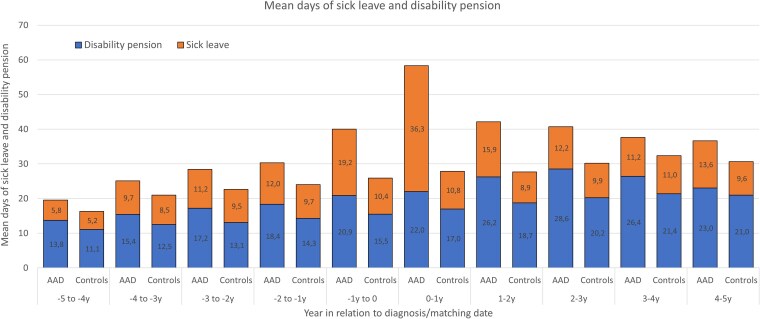
Mean days of sick leave and disability pension among incident patients with autoimmune Addison's disease (AAD) in Sweden between 2003 and 2019 and matched comparators, from 5 years before to 5 years after diagnosis.

Further analysis distinguished lost workdays due to sick leave from those due to disability pension. In the year prior to diagnosis, patients with AAD averaged 19.2 days of sick leave (vs 10.4 days in comparators) and 20.9 days of disability pension (vs 15.5 days). One year following diagnosis, sick leave rose to 36.3 days (vs 10.8), whereas disability pension remained almost unchanged at 22 days (vs 17.0). A full breakdown of these components is shown in Fig. 3.

**Figure 4.**
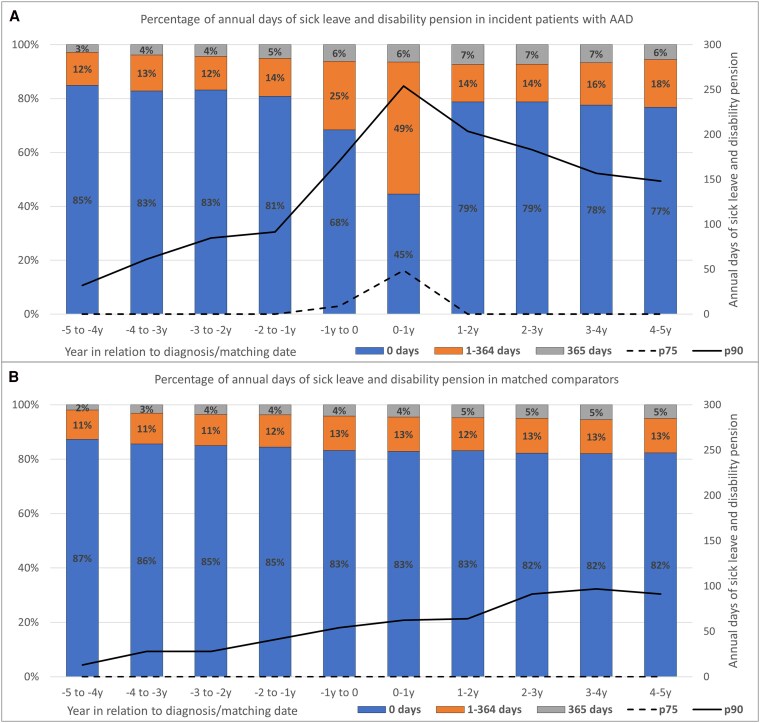
Percentage of annual days of sick leave and disability pension among A, incident patients with autoimmune Addison's disease (AAD) in Sweden between 2003 and 2019 and B, matched comparators, from 5 years before to 5 years after diagnosis. Sick leave refers to continuous periods of absence exceeding 14 days. Shorter episodes are not recorded in the registers.

Importantly, the proportion of individuals without any registered work absence (>14 days) declined from 68% among patients (vs 83% in comparators) the year before diagnosis to 45% (vs 83%) the year after diagnosis (Fig. 4).

### Taxable Earnings

**Figure 5.**
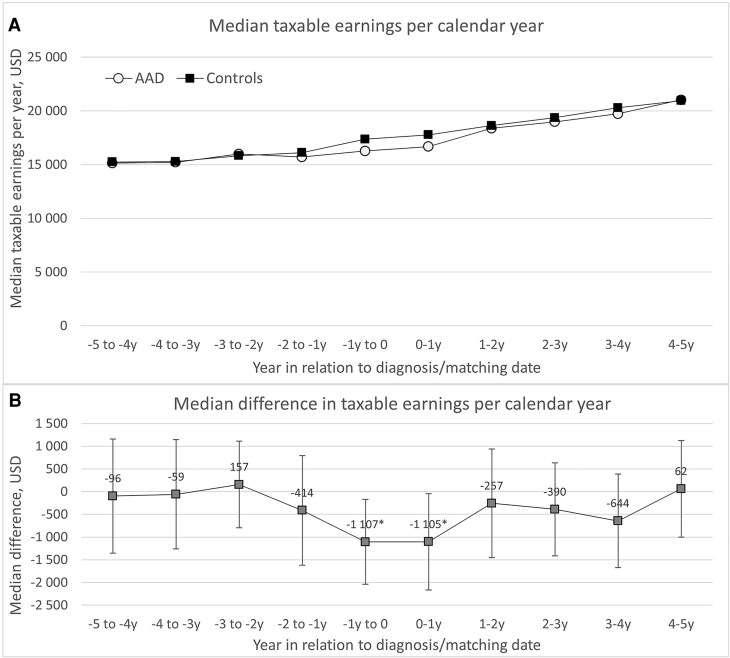
A, Median taxable earnings per year and B, median difference in taxable earnings per year among incident among incident patients with autoimmune Addison's disease (AAD) in Sweden between 2003 and 2019 and matched comparators, from 5 years before to 5 years after diagnosis. All values of taxable earnings are adjusted for inflation to 2024. *P less than .05. Abbreviations: AAD, autoimmune Addison's disease; USD, United States dollars.

Patients with AAD had significantly lower median taxable earnings compared to their matched comparators both the calendar year before and after diagnosis. The calendar year prior to diagnosis, the median taxable income was $16 259 among patients with AAD and $17 367 among comparators, yielding a median difference of $−1107 (95% CI, $−2042 to $−173; *P* = .02). The calendar year following diagnosis, median earnings were $16 676 in patients with AAD vs $17 769 in comparators, with a similar median difference of $−1105 (95% CI, $−2166 to $−43; *P* = .04) (Fig. 5).

### Disposable Income

**Figure 6.**
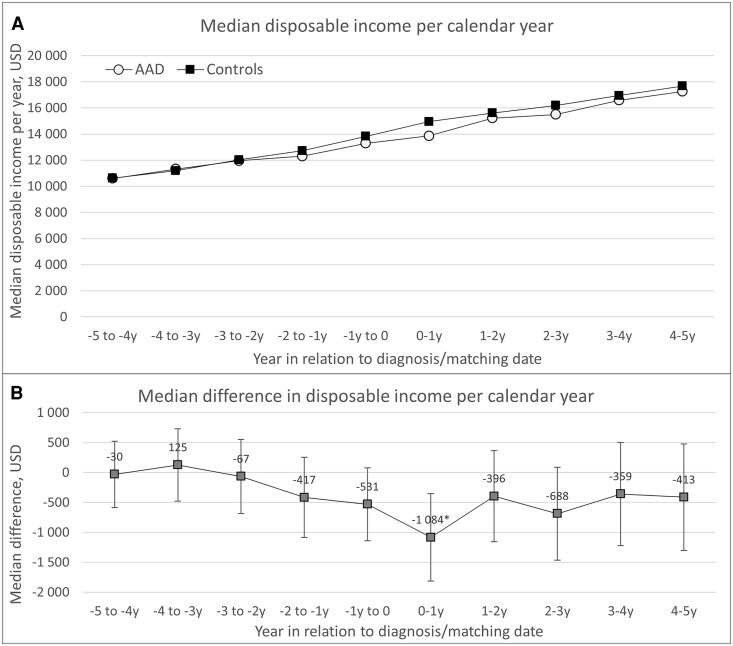
A, Median disposable income per year and B, median difference in disposable income per year among incident among incident patients with AAD in Sweden between 2003 and 2019 and matched comparators, from 5 years before to 5 years after diagnosis. All values of disposable income are adjusted for inflation to 2024. *P less than .05. Abbreviations: AAD, autoimmune Addison's disease; USD, United States dollars.

Patients with AAD had significantly lower median disposable income compared to matched comparators in the calendar year following diagnosis. During this period, the median disposable income was $13 860 in the AAD group and $14 944 in the comparator group, corresponding to a median difference of $−1084 (95% CI, $−1813 to $−354; *P* = .004) (Fig. 6).

### Subgroup Analyses

Across all outcomes, subgroup analyses showed patterns consistent with the overall findings, with no single subgroup emerging as a clear driver of the associations (Supplementary Figs. S1-S4 (41) and Supplementary Tables S4 and S5 (41)).

For work loss, the largest numerical differences 1 year after diagnosis were observed in women (35.7 days; 95% CI, 25.2-46.2 days), patients aged 40 to 62 years (36.2 days; 95% CI, 22.9-49.6 days), and those with concomitant type 1 diabetes (48.1 days; 95% CI, 24.5-71.7 days). Patients without type 1 diabetes displayed a pattern similar to the overall cohort, with work loss peaking at 28.0 days (95% CI, 20.1-35.9 days).

For taxable earnings, the most notable differences were seen in patients aged 40 to 62 years, with a median reduction of $–1565 (95% CI, $−3009 to $−120; *P* = .03) in the year before diagnosis and $−1770 (95% CI, $−2917 to $−624; *P* = .002) in the year after diagnosis. A similar pattern was found for disposable income, with the same age group showing significantly lower levels in both periods. While these subgroup patterns align with the overall results, the overlapping CIs indicate that the observed numerical differences should be interpreted with caution.

### Additional Analyses

At 5 years after the index date, the prevalence of autoimmune thyroid disease was 44.4% in patients with AAD compared with 4.5% in matched comparators, consistent with previous findings (20, 35).

During the original 5-year follow-up period, the hazard ratio for disability pension was 1.74 (95% CI, 1.01-2.99) (Supplementary Fig. S5 (41)). Extending the follow-up to 15 years after diagnosis increased the hazard ratio to 2.17 (95% CI, 1.42-3.31) (Supplementary Fig. S6 (41)).

## Discussion

This is the first longitudinal study to assess work loss and income in individuals with AAD, revealing significantly increased work loss and reduced income compared to matched population controls, particularly around the time of diagnosis. Importantly, work loss was largely driven by minority of individuals with AAD, while most patients were able to work or required only shorter periods of sick leave (<14 days) around diagnosis.

A 2022 review by Mira et al (42) highlighted a clear link between lower socioeconomic status (education, income, and wealth) and the progression of multiple chronic conditions in adulthood. Substantial evidence links wide income disparities to adverse health and social outcomes (43), underscoring the need for targeted public health strategies to address social inequalities in multimorbidity (44). This is particularly relevant given that AAD is frequently associated with other chronic autoimmune diseases.

Our findings align with and expand on earlier research demonstrating reduced work capacity among individuals with AAD. Løvås et al (21) were among the first to report that 26% of patients with AAD in Norway were work-disabled, with the largest proportion (50%) observed in patients aged 40 to 67 years with autoimmune polyendocrine syndrome type 2 (APS-2). Subsequent studies have similarly shown unemployment ranging from 14% to 19% in this population (22-24). More recently, a study identified an increased financial burden among individuals with adrenal insufficiency (25).

In our previous cross-sectional study, 23.1% of patients with AAD received financial compensation in 2019—13.1% for sick leave and 11.3% for disability pension—with only disability pension significantly higher than in comparators (20). In that study, long disease duration (>10 years) was associated with a significant reduction in work ability, mainly driven by disability pension. No increase in work disability was observed for 5 to 10 years' duration, while less than 5 years remained difficult to interpret. Taken together with the present findings (see Supplementary Fig. S6 (41)), the overall picture suggests an early decline in work ability—primarily due to sick leave—followed by partial recovery, and later a renewed decline over time, increasingly driven by disability pension.

Information on the specific occupations of study participants was not available. However, educational level may serve as an indirect indicator of occupational type, with individuals having shorter education typically engaged in manual labor (blue-collar work), while those with higher education are more often employed in office-based roles (white-collar work) (45, 46). Previous research suggests that individuals with higher education are more likely to return to work following extended sickness absence (47). In this study, patients with less than or equal to 9 years of education had numerically more lost workdays, whereas those with 13 or more years had fewer (Supplementary Fig. S3 (41)). The limited number of patients with AAD with short education (see Table 1) reduced the statistical power. Therefore, further comparisons across educational levels were not pursued.

Fatigue, along with cognitive and mood disturbances commonly reported by individuals with AAD (10, 11, 25, 48, 49), are known to negatively affect work ability (50, 51). Findings from previous studies on quality of life in patients with adrenal insufficiency suggest that, overall, women tend to report lower physical and mental health scores (25, 49), whereas results among older individuals show greater variability (23, 25, 49). Our results align with earlier research on these subgroups. National data indicate that, in Sweden, women are more likely than men to receive sick leave and disability benefits (52). This pattern was mirrored in our cohort: 1 year post diagnosis, the difference in lost workdays compared with matched comparators was 35.7 days among women and 22.9 days among men. Individuals aged 40 to 62 years appeared to have more lost workdays than those aged 20 to 39 years. However, as only working-age individuals were included, older populations were not evaluated.

Our findings suggest that the co-occurrence of type 1 diabetes is associated with greater work loss, particularly 1 year after AAD diagnosis. Previous research has established that type 1 diabetes negatively affects quality of life (22), employment status (53-55), income (54-57), absenteeism (55, 57, 58), and overall productivity (57-59) . For example, a Swedish study by Persson et al (55) reported lower employment rates among individuals with type 1 diabetes compared to controls (75.8% vs 80.4% for women; 83.8% vs 85.4% for men), along with a higher likelihood of receiving sickness benefits among women with diabetes (31% vs 19%; *P* < .001). In our previous cross-sectional study, we observed increased work loss and reduced income in 2019 among patients with AAD and comorbid type 1 diabetes (20). However, the combined effect of AAD and type 1 diabetes on work ability remains largely unexplored. Our findings suggest a possible synergistic effect on work loss and income, though this could not be formally assessed due to the low prevalence of type 1 diabetes among comparators. Results for patients without type 1 diabetes were consistent with the overall cohort (see Supplementary Fig. S4 (41) and Supplementary Tables S4 and S5 (41)).

In this study, patients with AAD experienced increased work loss from 1 year before to 3 years after diagnosis, while reductions in income were limited to the year immediately before and after diagnosis, reflecting the buffering effect of Sweden's welfare system. However, in countries with less comprehensive social support, the socioeconomic burden of AAD could be even greater, potentially deepening health and social disparities. In an era in which personalized and precision medicine is highly valued, these findings emphasize the need to support patients with AAD not only in achieving clinical stability but also in safeguarding their socioeconomic well-being. Identifying the particularly vulnerable subgroup of patients that experience considerable work-loss early in the course of their disease, and providing timely medical and psychosocial support, remains a challenge for clinicians but could potentially reduce the socioeconomic effect of the disease around the diagnostic period.

### Strengths

The primary strengths of this study include its population-based cohort design, and the use of a large, nationally representative dataset derived from linkage of high-quality Swedish registers with excellent internal validity and coverage. This design enabled robust comparisons with the general population and facilitated detailed subgroup analyses. Moreover, the prospective collection of data, independent of exposure status (AAD), reduced the potential for selection and information bias. The inclusion of a large, well-defined cohort of patients with AAD, despite the rarity of the disease, improved precision and statistical power. Restricting the cohort to individuals with AAD strengthened the internal validity of the study by ensuring a more homogeneous population with respect to disease etiology. The extended study period yielded a substantial number of outcome events, allowing for a comprehensive assessment of the socioeconomic consequences of the disease over time. Finally, the study's nationwide scope enhances its external validity.

### Limitations

The effect of health conditions or caregiving responsibilities on employment can be evaluated by assessing work productivity loss, typically comprising 3 main components: absenteeism, presenteeism, and changes in employment status (2). Due to the register-based design of this study, presenteeism—defined as reduced productivity while at work—could not be evaluated, as this would require direct data collection through interviews or questionnaires. However, our study provides reliable data on absenteeism and, to some extent, employment status changes.

Another limitation of this study is the absence of data on the specific medical or occupational causes of sick leave and disability pension. While educational level may serve as a partial proxy for occupational category and labor market sector, detailed information on these variables was not available. Additionally, the register used in this study captures only sick leave episodes exceeding 14 days, leading to an underestimation of total work loss. Therefore, potential associations between AAD and brief sick leave episodes lasting fewer than 14 days could not be evaluated. This limitation is particularly relevant as income could be affected since employers compensate 80% of the average weekly salary for shorter absences.

Data from the SAR were available only from 2008 onward, rather than from the study's inception. To ensure comparability, AAD cases and controls were matched based on sex, age, county of residence, calendar year, and level of education, allowing for the comparison of sociodemographically similar groups. The presence of residual confounding remains a potential limitation. Finally, individual factors such as educational or occupational choices, lower negotiated wages, informal or unreported employment, limited career advancement, or preferences for part-time work may have influenced the observed outcomes.

Substantial variation in social security systems and labor market regulations across countries may limit the generalizability of our findings to settings with different social and employment policies. However, because welfare provisions and labor regulations were consistent both for patients and comparators within the Swedish context, the observed group differences are likely to be internally valid and meaningful.

### Conclusion

In this nationwide cohort study, individuals with AAD exhibited increased work loss beginning 1 year prior to diagnosis and persisting for at least 3 years thereafter. A significant reduction in income was also observed in the calendar year preceding and following diagnosis. At the same time, it is reassuring that income appeared to normalize after the first year and that excess work loss was mainly concentrated to the years immediately surrounding the diagnosis. In less comprehensive welfare systems, the socioeconomic effect of AAD may be greater and exacerbate health and social inequalities. To fully understand the global effect of AAD on socioeconomic outcomes, further longitudinal studies in diverse health-care and welfare contexts are warranted, including efforts to better identify socioeconomically vulnerable patients.

## Data Availability

This study is based on Swedish national register data, which are subject to legal restrictions and therefore not publicly accessible. Pseudonymized data may be made available from the corresponding author on reasonable request.

## Abbreviations

AAD: autoimmune Addison's disease
ATC: Anatomical Therapeutic Chemical
ICD: International Statistical Classification of Diseases and Related Health Problems
LISA: Swedish Longitudinal Integrated Database for Health Insurance and Labor Market Studies
MIDAS: Micro Data for Analysis of the Social Insurance Database
NPR: Swedish National Patient Register
PDR: Swedish Prescribed Drug Register
SAR: Swedish Addison Register
SEK: Swedish kronor
TPR: Total Population Register
